# Notch Intracellular Domain Plasmid Delivery via Poly(Lactic-Co-Glycolic Acid) Nanoparticles to Upregulate Notch Pathway Molecules

**DOI:** 10.3389/fcvm.2021.707897

**Published:** 2021-09-28

**Authors:** Victoria L. Messerschmidt, Uday Chintapula, Aneetta E. Kuriakose, Samantha Laboy, Thuy Thi Dang Truong, LeNaiya A. Kydd, Justyn Jaworski, Zui Pan, Hesham Sadek, Kytai T. Nguyen, Juhyun Lee

**Affiliations:** ^1^Department of Bioengineering, University of Texas at Arlington, Arlington, TX, United States; ^2^Department of Internal Medicine, University of Texas Southwestern Medical Center, Dallas, TX, United States; ^3^College of Nursing and Health Innovation, University of Texas at Arlington, Arlington, TX, United States

**Keywords:** Notch signaling, PLGA, nanoparticles, gene delivery, non-viral transfection

## Abstract

Notch signaling is a highly conserved signaling system that is required for embryonic development and regeneration of organs. When the signal is lost, maldevelopment occurs and leads to a lethal state. Delivering exogenous genetic materials encoding Notch into cells can reestablish downstream signaling and rescue cellular functions. In this study, we utilized the negatively charged and FDA approved polymer poly(lactic-co-glycolic acid) to encapsulate Notch Intracellular Domain-containing plasmid in nanoparticles. We show that primary human umbilical vein endothelial cells (HUVECs) readily uptake the nanoparticles with and without specific antibody targets. We demonstrated that our nanoparticles are non-toxic, stable over time, and compatible with blood. We further demonstrated that HUVECs could be successfully transfected with these nanoparticles in static and dynamic environments. Lastly, we elucidated that these nanoparticles could upregulate the downstream genes of Notch signaling, indicating that the payload was viable and successfully altered the genetic downstream effects.

## Introduction

Notch signaling is highly conserved cell signaling pathway, which is involved in diverse embryonic organs or tissue development as well as regeneration (1–10). Notch signaling regulates cell-fate determination during activation by signal sending and receiving, affected through ligand-receptor crosstalk. During the cell-fate decisions in cardiac (8, 11, 12), neuronal (13–15), immune (16, 17), and endocrine (18, 19) development, the Notch signaling pathway acts as a key regulator of cell proliferation and differentiation (2, 4, 20). Notch receptors are single-pass transmembrane proteins composed of functional Notch extracellular domain (NECD), transmembrane (TM), and Notch intracellular domains (NICD). Notch receptors are processed in the endoplasmic reticulum and Golgi apparatus within the signal-receiving cell, through cleavage and glycosylation, generating a Ca^2+^-stabilized heterodimer composed of NECD non-covalently attached to the transmembrane NICD inserted in the membrane called S1 cleavage.

**Figure 1:**
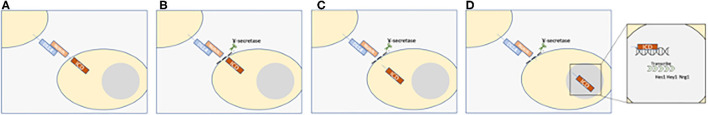
Notch signal activation. **(A)** Notch Receptor and DLL4 ligand bind together. **(B)** γ-secretase cleaves the Intracellular domain (ICD) from the extracellular domain. **(C,D)** The ICD is released and travels into the nuclease. (**D** inset) The ICD binds onto the DNA as a transcription factor to transcribe downstream Notch genes such as Hes1, Hey1, and Nrg1.

Regulation of arteriovenous specification and differentiation in both endothelial cells and vascular smooth muscle cells are also involved in Notch signaling including regulation of blood vessel sprouting, branching during normal and pathological angiogenesis, and the physiological responses of vascular smooth muscle cells (4, 6, 7, 21–23). Defects in Notch signaling also cause inherited cardiovascular diseases, such as Left Ventricular Non-compaction and Alagille syndrome (4, 7, 22). In endothelium, Delta-like ligand 4 (DLL4) is one of main ligands to send a signal to Notch in the adjacent cell (4, 6) (Figure 1A). This in turn signals the surrounding cells to determine the cell-fate (4). Once Notch is activated, the NICD is cleaved by γ-secretase and translocated into the nucleus (Figures 1B,C). Here, the NICD binds directly to the DNA, physically moving corepressors and histones, recruiting coactivators, and activating gene transcription (2, 4, 6) (Figure 1D).

When a disruption in this Notch pathway occurs, either by chemical or genetic means, it causes developmental malformations. For example, significant reduction of Notch signaling causing cardiac trabeculation is usually associated with deficient compaction in the ventricle (6, 24). It has been shown that lack of cardiac trabeculation results in the inability to dissipate the kinetic energy, resulting in a malformed heart due to a decrease in Notch related signaling (24, 25). Interestingly, when given NICD mRNA injection treatment, the heart function—including end diastolic function, end systolic function, stroke volume, and ejection fraction—were all partially or fully restored by rescuing downstream Notch signaling (25, 26). Regardless of whether the defect comes from the γ-secretase's inability to cleave the NICD, or if the native NICD is defective and unable to pass through the nucleus, by providing NICD mRNA to the cell it partially rescued the trabeculation. Similarly, when Notch signaling is inhibited from NICD cleavage or NICD translocation into the nucleus, Notch related downstream genes can rescue the feedback loop of Notch pathway (24). These data demonstrate the possible impact of spatiotemporal NICD treatment for therapeutic approach to rescue Notch signaling.

Traditionally, retroviruses or liposomes have been used to deliver cDNA plasmids (2, 27–29). These methods have various benefits such as DNA protection and DNA viability, but also have limitations of non-specific delivery, stability after formulation, or host immune responses (30, 31). Therefore, many groups are attempting to deliver the genetic materials such as cDNA plasmids via nanoparticles to mitigate these negative effects. Various polymers have been used for gene delivery (32–37). Cationic polymers have been used extensively to deliver genetic materials, as DNA condenses quickly on the oppositely charged positive polymer. These polymers can be synthetic or organic and usually include polyethylenimine (38, 39), polyamidoamine (40, 41), chitosan (42, 43), and cationic proteins (44), or peptides. However, the drawbacks of these highly positively charged polymers are mainly due to its toxicity (30, 31) and often require extensive surface modifications to alleviate those effects (31). Poly(lactic-co-glycolic acid) (PLGA), an FDA-approved biodegradable polymer (45), is a negatively charged polymer that has been extensively used for cancer treatment (46–49). More recently, PLGA has been used to load both hydrophilic and hydrophobic materials such as cDNA plasmids (33) and RNAs (50), proteins (51–53), dyes (54), and drugs (55).

In this study, we developed the PLGA nanoparticles encapsulating plasmids containing NICD for upregulation of Notch pathway molecules in cultured HUVECs. Using a flow chamber mimicking the *in vivo* circulation system, we evaluated the toxicity, stability, and compatibility in blood of the PLGA nanoparticles and our data suggested that we have here demonstrated NICD cDNA plasmid in the PLGA nanoparticles could upregulate Notch pathway molecules.

## Methods

### Nanoparticle Synthesis and Conjugation

Poly(D, L-lactide-*co*-glycolic acid) nanoparticles (PLGA, 50:50, Akina Inc., West Lafayette, IN, USA) of two different molecular weights including 55–65 kDa [High Molecular Weight (HMW) Nanoparticles] and 5–10 kDa [Low Molecular Weight (LMW) Nanoparticles] were fabricated by a standard double emulsion method as previously described (36). In brief, PLGA was dissolved in chloroform (Sigma-Aldrich, St. Luis, MO, USA) at a 20 mg·mL^−1^ concentration. Following which, the water phase with 1% (w/w) rhodamine B (Rh B) was added to the oil phase dropwise under stirring and sonicated. The primary emulsion is then emulsified into 5% (w/v) Poly(vinyl) Alcohol (PVA, 13 kDa, Sigma-Aldrich) solution and then sonicated at 40 Watts for 5 min (30 s off every 1 min). Nanoparticles were then collected via centrifugation at 15,000 RPM for 15 min, then lyophilized until completely dry. Coumarin-6 loaded PLGA nanoparticles were prepared to track the nanoparticles' interaction with the cells. For this, coumarin-6 was added into the oil phase at a ratio of 1:100 with respect to the amount of PLGA used during the nanoparticle synthesis. Rh B loaded nanoparticles were exclusively used to study model drug release kinetics.

TetO-FUW-NICD was a gift from Rudolf Jaenisch (Addgene plasmid #61540) and pCAG-GFP was a gift from Connie Cepko (Addgene plasmid #11150). pCAG-GFP or TetO-FUW-NICD loaded HMW nanoparticles were also prepared based on the same standard double emulsion method with slight modifications according to past literature (56). In brief, 250 μg of plasmid was diluted in 5% glucose solution to 200 μL which was then emulsified into 0.5 mL of 5% (w/v) PLGA solution in chloroform using a probe sonicator at 40 W energy output for 15 s to form primary water/oil emulsion. The primary emulsion was then emulsified into 3 mL of 4% (w/v) PVA solution by sonication and later dropped into 7.5 mL of 0.3% (w/v) PVA solution while stirring. The final mixture was then stirred for 3 h at room temperature and particles were collected by centrifugation. Nanoparticles were then lyophilized until completely dry before use.

PLGA nanoparticles were conjugated either with anti-EGFL7 antibody (ab92939, Abcam) or anti-Tie2+Tie1 antibody (ab151704, Abcam) via EDC-NHS chemistry as described elsewhere with modification (36). In brief, nanoparticles were suspended in 0.1M MES buffer at a concentration of 2 mg/ml. Following which, 120 mg of EDC and 150 mg of NHS was added into the solution. After 2 h of incubation at room temperature, nanoparticles were collected by centrifugation and resuspended in PBS (2 mg/ml). Twenty five microliter of antibody solution was added into nanoparticles solution and incubated overnight at 4°C. The supernatant was used to determine the antibody conjugation efficiency using Bradford assay following manufacturers' instructions. Pellets were resuspended in DI water, freeze-dried, and stored for use.

### Characterization and Stability of Nanoparticles

To determine the size and surface charge, nanoparticle suspension was added to a transparent cuvette and was then inserted into the ZetaPALS dynamic light scattering (DLS) detector (NanoBrook 90Plus PALS, Brookhaven Instruments, Holtsville, NY) as previously described (36). Scanning electron microscopy (SEM, Hitachi S-3000N, Hitachi, Pleasanton, CA) was used to visualize the morphology of nanoparticles. Briefly, 50 μl of the nanoparticle suspension air-dried on a coverslip was silver sputter-coated and inserted into the SEM instrument. To determine the *in vitro* stability, nanoparticles were suspended in saline (0.9% Sodium Chloride, NaCl, Crystalline, Fisher Scientific, Hampton, NH, USA) or Vasculife VEGF basal cell media with 10% Fetal Bovine Serum (LL-0003, Lifeline Cell Technologies) and incubated at 37°C for 48 h. Particle size was measured on predetermined time points using DLS as described earlier. The stability of the nanoparticles was represented as the percentage change of nanoparticle size measured at each time point with respect to initial particle size according to the following equation:


(1) $$\begin{array}{l} Size\;\left(\%\right)=\frac{{Nanoparticle\;Size}_{t=t_{0}}}{{Nanoparticle\;Size}_{t=0}}*100 \end{array}$$

### Loading and Release Studies

The encapsulation efficiency of entrapped reagent including, pCAG-GFP or TetO-FUW-NICD, within PLGA nanoparticles was determined based on indirect loading analysis. Briefly, the un-loaded reagent in the supernatant (PVA solution) following the nanoparticle synthesis, was used to calculate the encapsulation efficiency (Equation 2). The amount of plasmid was determined using Picogreen DNA assay (#E2670, Promega, Madison, WI) following the manufacturers' instructions.


(2) $$\begin{array}{l} Encapsulation\;Efficiency\;\left(\%\right)=\frac{Plasmid\;initially\;added-plamid\;in\;supernatant}{Plasmid\;initially\;added}*100 \end{array}$$

For *in vitro* plasmid release studies, solutions of pCAG-GFP or TetO-FUW-NICD plasmid-loaded nanoparticles were prepared in 1X PBS at a concentration of 1.5 mg/mL. At predetermined time points, the samples were centrifuged at 12,000 RPM for 5 min. The supernatant was then collected and stored at −20°C for further analysis. Pellet was again resuspended in fresh 1 mL of PBS solution and incubated until next time point. Four replicates were used for analysis. For analysis, the plasmid solutions were incubated with Nb.Bsmi nicking enzyme (R0706S, New England Biolabs) for 60 min at 65°C in NEBuffer 3.1. The enzyme was then inactivated for 20 min at 80°C. The nicked plasmid supernatant was analyzed for plasmid release using the Picogreen DNA assays. The plasmid standards were made to determine the cumulative percentage of plasmid release over time.

### *In vitro* Compatibility of Nanoparticles

HUVECs were cultured in M199 media (M4530, Sigma-Aldrich) supplemented with Vasculife VEGF LifeFactors kit (LS-1020, Lifeline Cell Technologies) up to passage 7 in a 5% CO_2_ environment. To determine the compatibility of nanoparticles, HUVECs were seeded in 96 well plates at seeding density of 8,000 cells/well and cultured overnight. HMW nanoparticles and LMW nanoparticles of various concentrations (25, 50, 100, 250, 500, 1,000 μg mL^−1^) were prepared in complete M199 media and added to the cells. After 24 h of incubation at 37°C, the nanoparticle containing media was removed, and cells were carefully washed with 1X PBS. The cellular viability was then determined using MTS assays per manufacturer's instructions.

In addition, HMW nanoparticles and LMW nanoparticles compatibility was evaluated using human whole blood, to determine hemolysis and whole blood clotting kinetics assay as previously mentioned. For these studies, whole blood was drawn from healthy adult volunteers into acid citrate dextrose anticoagulant tubes (ACD, Solution A; BD Franklin Lakes, NJ). Consent from the volunteers was obtained prior to the blood collection, and all the procedures strictly adhered to the IRB standards approved at the University of Texas at Arlington.

To perform whole blood clotting study, the blood was initially activated by adding 0.01 M of calcium chloride (Sigma). Following which, 50 μL of activated blood was added into 10 μL of saline diluted nanoparticle solution at concentration of 1 mg/mL and incubated for predetermined time points. At each time point, 1.5 mL of DI water was added to lyse the un-clotted blood and the absorbance of the supernatant was measured at 540 nm. Untreated blood served as a control. In the hemolysis study, nanoparticles were suspended in saline at the following concentrations (0, 10, 25, 50, 100, 250, 500, 1,000 μg·mL^−1^) and then incubated with 200 μL of saline-diluted blood for 2 h at 37°C. Following the incubation, the samples were centrifuged, and the absorbance of the supernatant was quantified at 545 nm. Untreated blood that was diluted with DI water and saline solution served as positive and negative controls, respectively. The percent hemolysis was calculated using the following equation:


(3) $$\begin{array}{l} \%=\;\frac{Abs_{sample}-Abs_{neg\;ctl}}{Abs_{pos\;ctl}-Abs_{neg\;ctl}}\times 100 \end{array}$$

### *In vitro* Cellular Uptake and Interaction of Nanoparticles

To determine the uptake of coumarin-6 loaded HMW- and LMW-PLGA nanoparticles by HUVECs, cells were seeded in 96 well-plates at a density of 8,000 cells/well. After overnight culture, nanoparticles of various concentrations 50, 100, 250, 500, 1,000 μg·mL^−1^ were added to the cells and incubated for 4 h in 37°C. Nanoparticles were then removed, cells were carefully washed with PBS solution and lysed using 1% Triton X-100. Fluorescence intensity measurement of nanoparticles in cellular lysate was quantified at a wavelength of 457 nm (excitation)/500 nm (emission) using a spectrophotometer. These measurements were analyzed against a nanoparticle standard. The measurements were further normalized with respect to the sample cellular protein amount as determined based on BCA assay (Thermofisher Scientific).

Similarly, interaction between antibody (anti-EGFL7 or anti-Tie2+Tie1) conjugated HMW nanoparticles loaded with coumarin-6 and HUVECs were also determined under static conditions. In brief, nanoparticle suspensions were treated with cells for 30 min and following which, cells were washed and lysed. Cellular lysate was used to determine the amount of nanoparticle attachment and internalization with HUVECs based on coumarin-6 fluorescence intensity. These fluorescence measurements values were then normalized with the total DNA content per sample using Picogreen DNA assays per manufacturer's instructions. In parallel, nanoparticle interaction with HUVECs were observed using a fluorescence microscope under FITC channel. The cells were counterstained using Nucblue (Invitrogen) to visualize the cell nuclei. To show the specificity of the optimal antibody to endothelial cells, HL-1 cells were cultured overnight in a 96-well plate overnight in Claycomb media supplemented with 10% FBS, 1% pen-strep, 0.1 mM Norepinephrine, and 2 mM of L-Glutamine. The following day, nanoparticles conjugated with anti-Tie2+Tie1 were added to the HL1 cells at 100, 250, 500, 1,000 μg·mL^−1^ for 4 h. After the incubation, cells were lysed, and fluorescence read under the same conditions. The fluorescence was normalized to DNA content.

In addition, the ability of a coumarin-6 loaded, antibody (anti-EGFL7 or anti-Tie2+Tie1) conjugated HMW nanoparticles to adhere and interact with HUVECs under physiological relevant flow condition was investigated. HUVEC's were seeded at 2^*^10^6^ cells/mL into μSlide VI^0.4^ channel and cultured overnight. Following the cell attachment, nanoparticles suspended in M199 media at a concentration of 200 μg/mL were perfused through the channels of the flow slide using Ibidi pump system at a shear stress of 5 dyne/cm^2^ for 30 min. Later, cells within the channels were fixed with paraformaldehyde solution and treated with Nucblue (Invitrogen) to stain cell nuclei. The cellular images were then taken using fluorescence microscope under FITC and DAPI channel to visualize the nanoparticles and nuclei, respectively. The fluorescence intensity of nanoparticles was later quantified using NIH ImageJ software and normalized by cell number.

To further prove our nanoparticle selectivity, we coated μ-Slide IV 0.4 (Ibidi, #80606) with 14.4 μg of bovine serum albumin (BSA), a 1:1 solution of Tie1 and Tie2 protein at 14.4 μg, or 1X PBS. The solutions were left at room temperature for 2 h. The remaining solution was washed off. Coumarin 6 nanoparticles were prepared as above, and conjugated with either BSA, anti-Tie2+Tie1, or were unconjugated. Nanoparticle media at a concentration of 250 μg/mL was flowed through at 5 dyne·cm^2^ for 15 min. The media was removed and washed with 1x PBS to remove unbound nanoparticles. The slides were imaged at 100 × to visualize the bound content. Using ImageJ, the intensity of the fluorescence was measured to quantitatively evaluate the binding.

### Plasmid Transfection

HUVECs were seeded 24 h prior to the transfection study at *n* = 4. The following day, Lipofectamine 3000 or no treatment were applied to the cells for 6 h. After the treatment, the cells were washed three times with 1X PBS and incubated until the next time point. HMW PLGA nanoparticles were prepared as described above. The nanoparticles at a concentration of 250 μg/mL were then applied to HUVECs for 6 h. The cells were then gently washed with 1X PBS three times and new media given. The cells treated with Lipofectamine, nanoparticles, or no treatment were then grown for 24, 48, or 72 h post transfection. Cells transfected with pCAG-GFP plasmid-loaded nanoparticles were imaged in a fluorescent microscope on FITC channel, nuclei were stained with Nucblu. The intensity of each fluorescent channel was measured via ImageJ. The data was then normalized by cell number, via Nucblu intensity, then normalized to the untreated cell group following the Equation 4.


(4) $$\begin{array}{l} Mean\;Correlated\;Total\;Cell\;Fluorescence=\;\left(GFP\;Intensity\right)/\left(NucBlu\;Intensity\right) \end{array}$$

Before loaded into nanoparticles, the quality and quantity of TetO-FUW-NICD plasmid were analyzed by digestion to ensure positive clones were used. Four biological repeats were carried out for each experiment.

### RT-PCR Data

Cells were first washed with 1X PBS two to three times. Then, 0.025% trypsin was added for 5 min at 37°C to allow cell detachment. The trypsin was then neutralized by adding media twice the volume of trypsin to the wells. The cells were collected, centrifuged at 150 × g for 5 min, and the supernatant discarded. The cells were then used to isolate the total RNA using the Aurum Total RNA Mini Kit (Biorad, #7326820) following the manufacturer's instructions. RNA concentration was determined via NanoDrop, by reading each sample 3 times. The total RNA was then used to synthesize 200 ng of cDNA using the iScript Synthesis Kit (Biorad, #1708890) following the manufacturer's instructions. PCR was conducted using the iTaq Universal SYBR Green Supermix (Biorad, #1725121) following manufacturer's instructions. The primer sequences for human mRNA are as follows: *Dll4* (Frd CTGCGAGAAGAAAGTGGACAGG, Rev ACAGTCGCTGACGTGGAGTTCA), *Hes1* (Frd GGAAATGACAGTGAAGCACCTCC, GAAGCGGGTCACCTCGTTCATG*), Hey1* (Frd ACCATCGAGGTGGAGAAGGA, Rev AAAAGCACTGGGTACCAGCC), *Notch1* Receptor (Frd GGTGAACTGCTCTGAGGAGATC, Rev GGATTGCAGTCGTCCACGTTGA), NICD (Frd ACCAATACAACCCTCTGCGG, Rev GGCCCTGGTAGCTCATCATC), and β-*Actin* (CGACAGGATGCAGAAGGAG, Rev ACATCTGCTGGAAGGTGGA).

### Western Blot

Cells were cultured in a 6-well plate overnight. The following day, nanoparticles loaded with NICD plasmid, nanoparticles loaded with NICD plasmid and conjugated anti-Tie2+Tie1, blank nanoparticles, or cell media were added to the culture. After an additional 24 h with treatment and shear stress, the media was removed, cells washed with 1x PBS, and lysed with radio-immunoprecipitation assay buffer supplemented with protease inhibitor cocktail (Roche). Protein concentrations were determined via the Pierce BCA Assay Kit (ThermoFisher). Antibodies against Notch1 (Invitrogen, MA5-32080), Hey1 (Abnova, H00023462-M02), Hes1 (OriGene, TA400013), and GAPDH (Proteintech, HRP-60004) were probed at suggested dilutions. Secondary antibodies conjugated with horseradish peroxidase were incubated and detected by enhanced chemiluminescence reagent (BioRad). The total protein of each well was measured using ImageJ's Gel Analysis. Similarly, each individual band was measured using the same technique, then normalized to the total protein amount.

### Statistical Analysis

All statistics were evaluated in the statistical program R. For the percent change in size, a one-way ANOVA was used to compare each sample to its' original size. A one-way ANOVA was also used to determine significance of nanoparticle uptake between anti-Tie2+Tie1 or anti-EGFL7 nanoparticles, antibody uptake in dynamic culture, nanoparticle dose study, and gene expression between static and dynamic culture. A two-sample *t*-test was used to compare the HMW to LMW in the cell viability and nanoparticle cellular interaction studies. Similarly, the gene expression was evaluated to compare dynamic culture at 12 dyne·cm^−2^ to static culture for each gene. All values where *p* < 0.05 were considered significant. *Post-hoc* Tukey tests were conducted if ANOVA results showed significance to determine differences between groups.

## Results

### Optimization of Nanoparticles Based on Molecular Weight of PLGA

**Figure 2:**
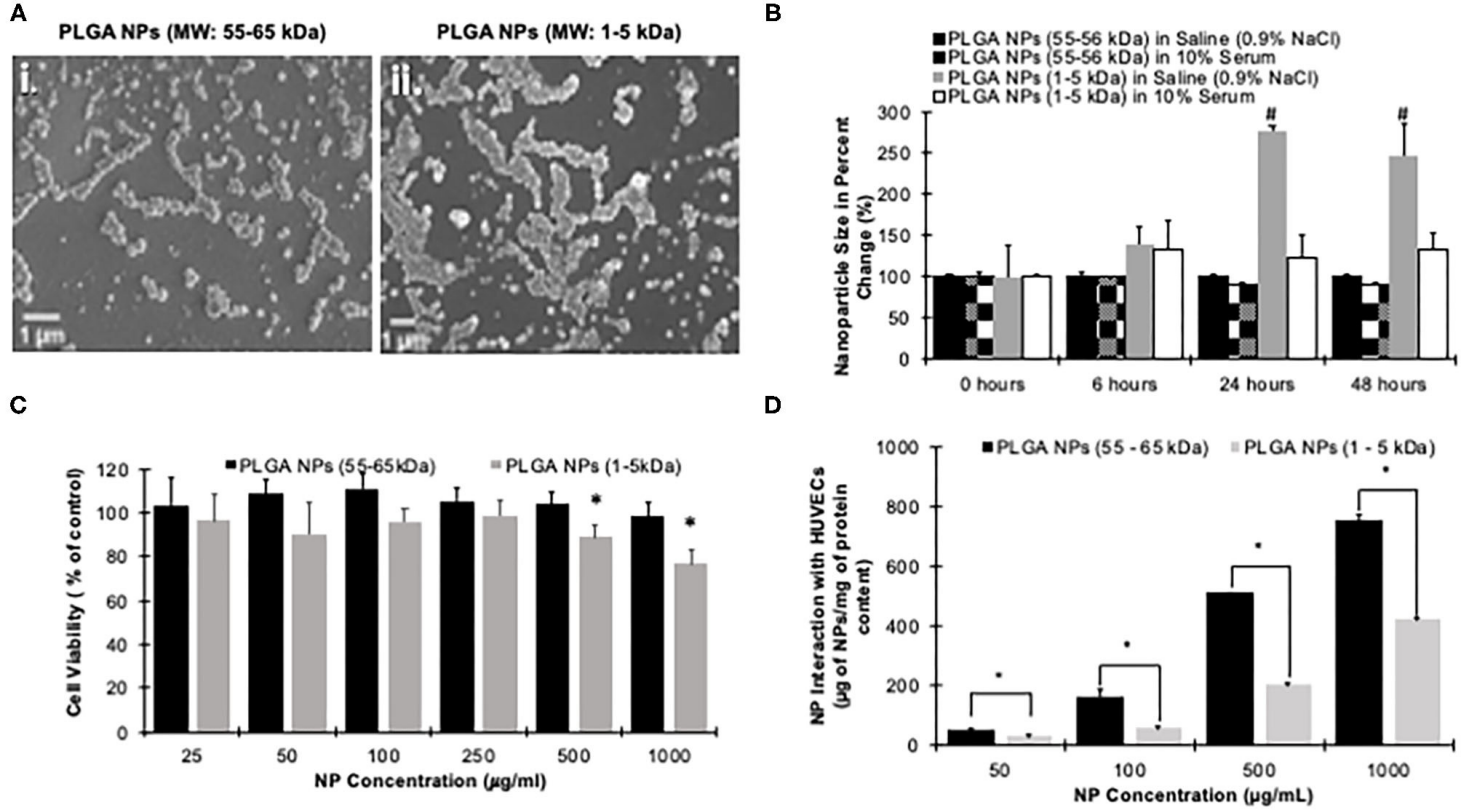
Characterization of PLGA nanoparticles. **(A)** SEM images of (i) PLGA at 55–65 kDa and (ii) PLGA at 1– 5 kDa nanoparticles. Aggregation occurs during the air-drying process due to the shrinking water droplet on the glass slide for taking this images. Scale bar is 1 μm. **(B)** Nanoparticles stability in saline (0.9% NaCl) or 10% Serum over time shows that the HMW PLGA nanoparticles' size is steady in both solutions, while the LMW PLGA nanoparticles vary in both the Saline and 10% Serum. Error bars denote standard error. #Indicates a significant difference from the samples' time point 0 (*p* < 0.05) evaluated via one-way ANOVA. **(C)** Cytocompatibility test comparing HMW to LMW PLGA nanoparticles. This shows at all concentrations the HMW PLGA nanoparticles are insignificantly toxic to the cells. The LMW nanoparticles are tolerated up to a concentration of 250 μg/mL. *Indicates a significant difference from 100% viability evaluated via two-way ANOVA (*p* < 0.05). **(D)** HUVEC uptake of both HMW and LMW PLGA nanoparticles shows HMW had significantly higher uptake than LMW at all tested concentrations. Data shown as mean + standard error. *Indicates a significant difference between HMW and LMW Nanoparticle uptake evaluated at each time point via a *t*-test (*p* < 0.05).

Before performing the cell study, nanoparticles were characterized based on their size, poly dispersity, and zeta potential (Table 1). The diameter of high molecular weight (HMW) PLGA nanoparticles, at 55–65 kDa, were smaller than the low molecular weight (LMW), 1–5 kDa, PLGA nanoparticles at 234 ± 90 and 246 ± 85 nm, respectively. The zeta potential, or surface charge of the nanoparticles, indicates the presence of the negatively charged carboxyl and hydroxyl groups present on the polymer. The HMW PLGA nanoparticles have a charge of −31 ± 3.4 mV, and the LMW PLGA nanoparticles have a charge of −29 ± 2.8 mV. The poly dispersity of both the HMW and LMW PLGA nanoparticles, 0.13 ± 0.05 and 0.08 ± 0.02, respectively, shows that the particles are uniformly dispersed. SEM images also indicated that both the HMW- and LMW-nanoparticles were uniformly dispersed and have spherical morphology (Figure 2A).

**Table 1:** PLGA nanoparticle physical attributes.

**PLGA nanoparticles**	**Size (nm)**	**Poly dispersity**	**Zeta potential (mV)**
MW: 55–65 kDa	234 ± 90	0.13 ± 0.05	−31 ± 3.4
MW: 1–5 kDa	246 ± 85	0.08 ± 0.02	−29 ± 2.8

Following *in vitro* stability studies using HMW- and LMW-nanoparticles in both saline (0.9% NaCl) and 10% serum, the nanoparticle percent size change was determined. Accordingly, the diameter of HMW nanoparticles were constant in both formulations over 48 h of incubation, which indicates the superior stability properties of HMW nanoparticles. On other hand, the size of LMW nanoparticles steadily increased over time and showed significant aggregation following their incubation with the saline solution at 24 h. In serum, the LMW nanoparticles increased in size, but was not significantly different (Figure 2B). This suggests that LMW nanoparticles may exhibit aggregation behavior following their suspension and/or administration. Then, the drug release kinetics were then compared between the two molecular weights using a model hydrophilic drug Rh B. High and low molecular weight nanoparticles were incubated in 1X PBS over a period to assess Rh B release kinetics. Both molecular weights of PLGA nanoparticles showed a burst release of Rh B dye with LMW releasing all the dye within 5 days and the HMW nanoparticles with a sustained release of over 20% by day 28 (Supplementary Figure 1).

To assess the cytocompatibility of nanoparticles, HUVECs were subjected to varying concentrations of both HMW and LMW nanoparticles. Across all tested concentrations, the HMW nanoparticles were all above 90% viability, while the LMW had >90% viability in only 25, 50, 100, and 250 μg/mL (Figure 2C). At both 500 and 1,000 μg/mL, the LMW nanoparticles were significantly lower at 88 ± 10 and 76 ± 13% viability, respectively (*p* < 0.05). The uptake of the nanoparticles was evaluated using HUVECs incubated with varying amounts of nanoparticles. At each tested concentration, the HMW nanoparticles had a significantly higher uptake compared to that of the LMW. Additionally, there is a trend showing a dose-dependent relationship between the number of nanoparticles applied, and the number of nanoparticles endocytosed by the cells (Figure 2D).

### Compatibility in Blood

**Figure 3:**
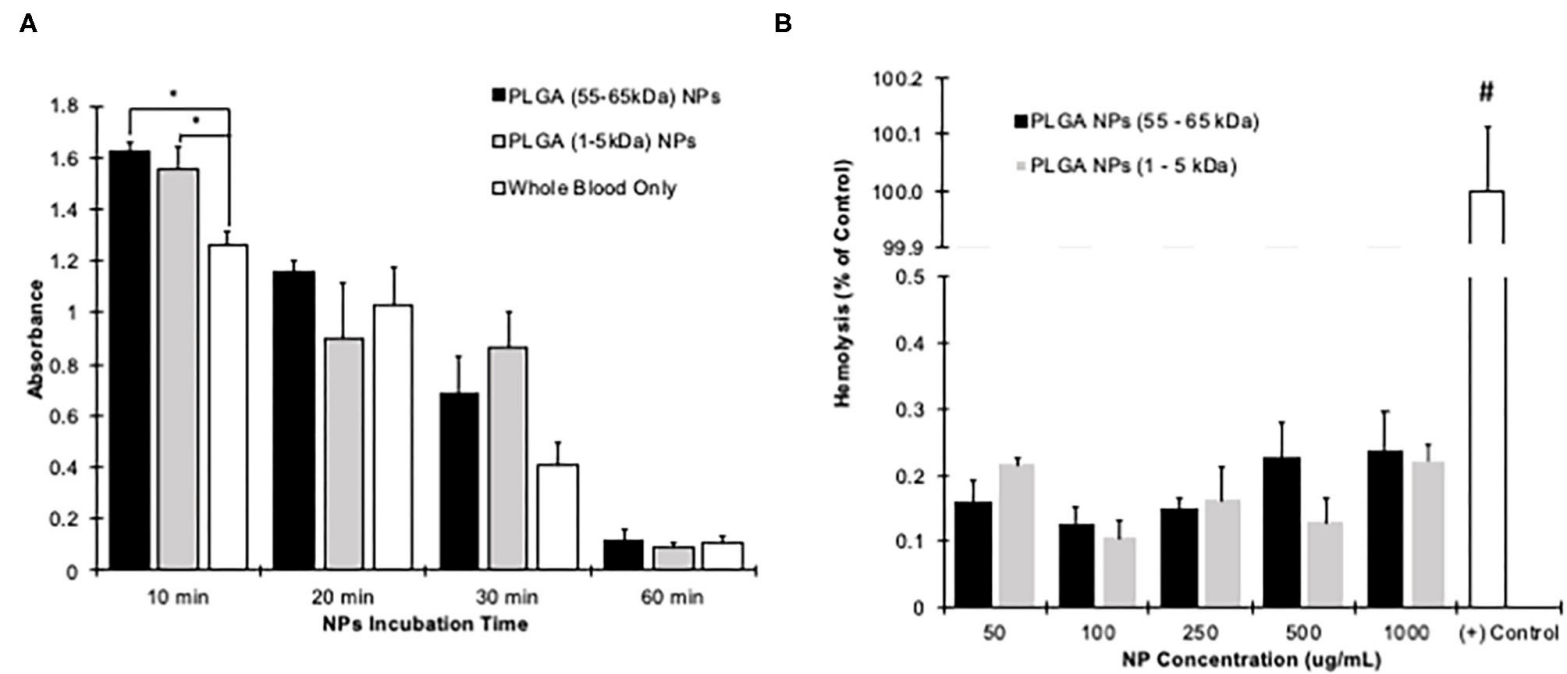
Hemocompatibility of PLGA nanoparticles. **(A)** Nanoparticles at a concentration of 1 mg/mL were subjected to human blood for up to 1 h. The clotting was significantly affected only during the first 10 min of nanoparticle exposure (*p* < 0.05). At all other time points, the nanoparticles did not affect the clotting ability of the human blood. Blood exposed to only air was kept as a control. *Denoted a significant difference with *p* < 0.05 evaluated via one-way ANOVA at each time point. **(B)** Nanoparticles were incubated with blood for 1 h. Compared to RO water treatment (Positive control), both nanoparticle groups had significantly less hemolysis at all tested concentrations. #Indicates that the Positive control is significantly higher than all other groups with *p* < 0.05 evaluated using a two-way ANOVA. All data is shown as mean + standard error.

To simulate the effect of nanoparticles on human blood, hemolysis and whole blood clotting tests were conducted. For whole blood clotting, the nanoparticles significantly affected the clotting cascade only during the first 10 min of exposure. Afterwards, the progress of blood clotting gradually reduced and there were no significant results compared to whole blood without exposing PLGA nanoparticles (Figure 3A). After 60 min, blood exposed to either HMW or LMW nanoparticles had great low supernatant absorbance, 0.1, similar to whole blood which indicates blood clot. Our results reflect those who have performed similar studies showing little red blood cell lysis or reduced clotting kinetics (57). Furthermore, the interaction between red blood cells and nanoparticles were evaluated by incubation with diluted blood to determine if hemolysis occurred. Compared to lysed cells as the positive control, both the HMW and LMW nanoparticles were significantly lower (<0.25%) in hemolysis (Figure 3B).

### Selection of Optimal Endothelial Target

For this study, HMW nanoparticles were used because the HMW has greater cell uptake and cell viability properties even though LMW nanoparticles have a rapid release profile. Anti-EGFL7 and Anti-Tie2+1 were conjugated to PLGA HMW nanoparticles and characterized. The nanoparticles conjugated with anti-EGFL7 increased to 249 ± 55 nm, while the nanoparticles conjugated with anti-Tie2+Tie1 are 243 ± 41 nm. Both antibody conjugations had a low poly dispersity, indicating that most of the nanoparticles were uniform in size. The antibodies changed the surface charge of the nanoparticles from −31 ± 3.4 to −23.5 ± 1.7 mV for anti-EGFL7 nanoparticles, and −31 ± 3.4 to −27.4 ± 1.8 mV for anti-Tie2+Tie1 nanoparticles. The antibodies had a conjugation efficiency of 59.6 ± 1.5 and 47.5 ± 1.2% for anti-EGFL7 conjugated nanoparticles and anti-Tie2+Tie1 conjugated nanoparticles, respectively (Table 2).

**Table 2:** Endothelial cell targeted PLGA nanoparticles.

**Antibody conjugated nanoparticles**	**Size (nm)**	**Poly dispersity**	**Zeta potential (mV)**	**Conjugation efficiency (%)**
Anti-EGFL7 nanoparticles	249 ± 55	0.21 ± 0.01	−23.5 ± 1.7	59.6 ± 1.5
Anti-Tie2+Tie1 nanoparticles	243 ± 41	0.19 ± 0.13	−27.4 ± 1.8	47.5 ± 1.2

**Figure 4:**
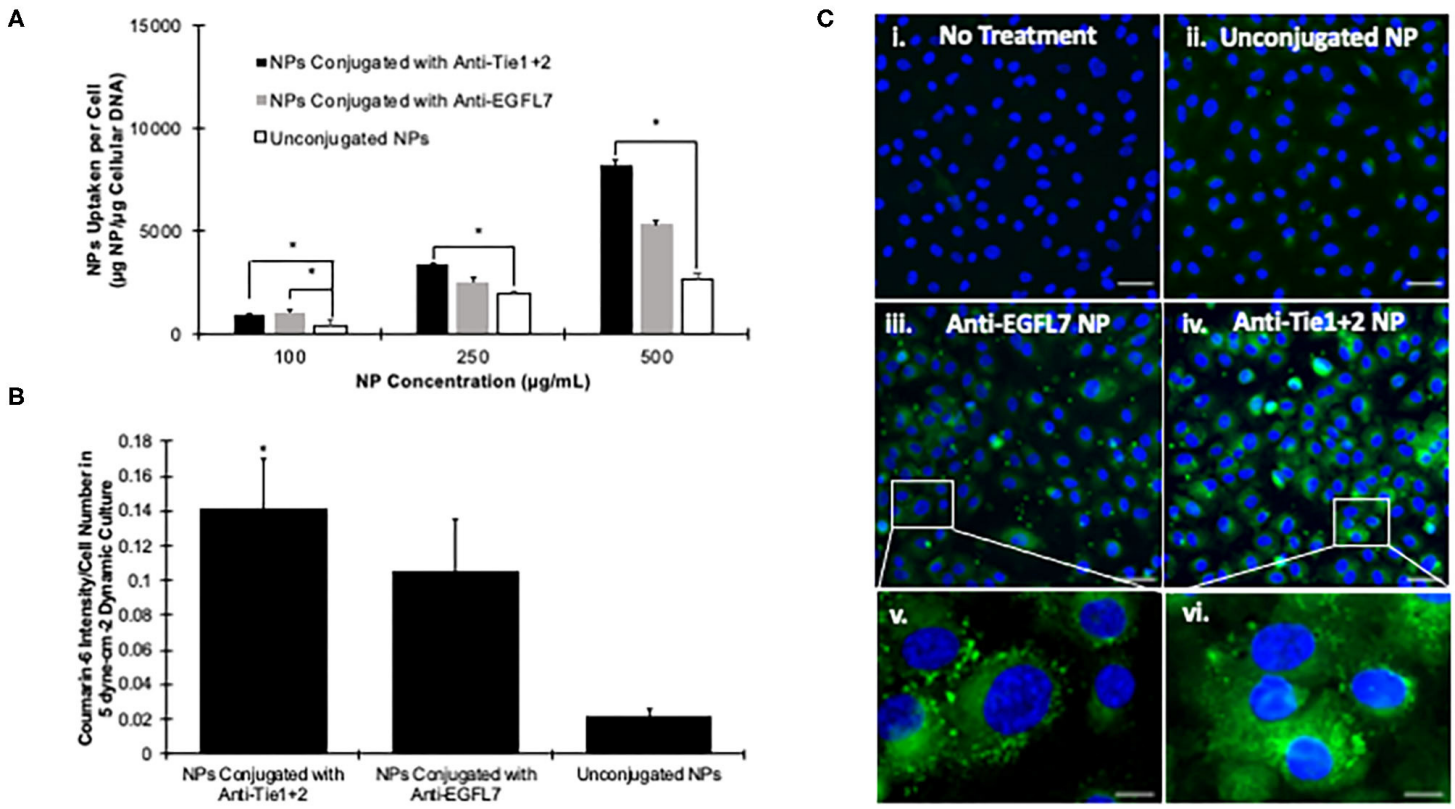
Targeting efficiency of antibody conjugated PLGA nanoparticles. **(A)** HUVEC Cellular uptake of endothelium specific anti-EGFL7, anti-Tie2+Tie1 conjugated nanoparticles or unconjugated nanoparticles after 4 h. *Indicates a significant difference via one-way ANOVA (*p* < 0.05). Data is shown as mean + standard deviation. **(B)** HUVEC Cellular uptake under 5 dyne·cm^−2^ of endothelium specific anti-EGFL7 and anti-Tie1+2 conjugated nanoparticles, or unconjugated nanoparticles. *Denotes a significant difference between anti-Tie2+Tie1 nanoparticles and unconjugated nanoparticles via one-way ANOVA (*p* < 0.05). Data is shown as mean + standard deviation. **(C)** Fluorescent images of HUVEC's incubated in flow culture of 5 dyne·cm^−2^ with (i) No Treatment, (ii) Unconjugated Nanoparticles, (iii) Anti-EGFL7 conjugated nanoparticles, (iv) anti-Tie1+2 conjugated nanoparticles. Scale bar = 20 μm. Zoomed in portions of HUVEC's incubated in flow culture at 5 dyne·cm^−2^ with (v) Anti-EGFL7 conjugated nanoparticles, and (vi) anti-Tie2+Tie1 conjugated nanoparticles. Scale bar = 5 μm.

Furthermore, we tested our antibody conjugated particles on their uptake abilities under static and physiological flow conditions. Under static conditions, we saw concentration-dependent uptake of nanoparticles by endothelial cells (Figure 4A). As the concentration of anti-Tie2+Tie1 conjugated nanoparticles increases, the rate of cellular uptake increases 3.5 and 8.4 folds from 100 to 250 and 500 μg/mL, respectively (Figure 4A). Similarly, anti-EGFL7 conjugated nanoparticles increase 2.4-folds and 5.1-folds from 100 to 250 and 500 μg/mL, respectively. Additionally, the unconjugated nanoparticles increase 5.2 and 7.3-fold from concentrations of 100 to 250, and 500 μg/mL, respectively. Additionally, antibody conjugated nanoparticles had a greater interaction with the cells compared to unconjugated ones. Coumarin-6 loaded HMW nanoparticles conjugated to either anti-EGFL7 or anti-Tie2+Tie1 supported the quantitative data (Supplementary Figure 2).

Compared to unconjugated nanoparticles, antibody conjugated nanoparticles to target endothelial cells show higher uptake efficiency although the diameter of nanoparticles were increased (Figure 4B). Tested with flow system, nanoparticles conjugated with anti-EGFL7 has significantly higher cellular uptake. However, nanoparticles conjugated with anti-Tie2+Tie1 were significantly higher in cellular uptake than that of anti-EGFL7 conjugated. With fluorescent imaging, we can visualize that under flow conditions at 5 dyne·cm^−2^, the antibody conjugated nanoparticles were able to be endocytosed into cells at a higher rate compared to unconjugated nanoparticles (Figure 4C). To further prove the targeting ability of our anti-Tie2+Tie1 nanoparticles, we tested their binding ability to different protein coatings. In addition to anti-Tie2+Tie1 coated nanoparticles, we compared BSA conjugated and unconjugated nanoparticles on their binding ability to Tie1/Tie2-coated, BSA-coated, or uncoated slides. The Tie2+Tie1 nanoparticles bound to the Tie2+Tie1-coated slides significantly higher than both the BSA conjugated nanoparticles and the unconjugated nanoparticles (Supplementary Figures 4A,B). Additionally, we cultured cardiomyocytes, HL1 cells, with anti-Tie2+Tie1 nanoparticles to investigate if the NP uptake was specific to endothelial cells. There was a significant difference at all tested concentrations between HUVECs and HL1 cells (Supplementary Figure 4C). Due to the increase in cellular uptake of nanoparticles conjugated with anti-Tie2+Tie1 and its specificity toward Tie1/Tie2 coating and endothelial cells, this antibody was determined to be superior for endothelial targeting.

### Characterization of Plasmid Loaded PLGA Nanoparticles

**Figure 5:**
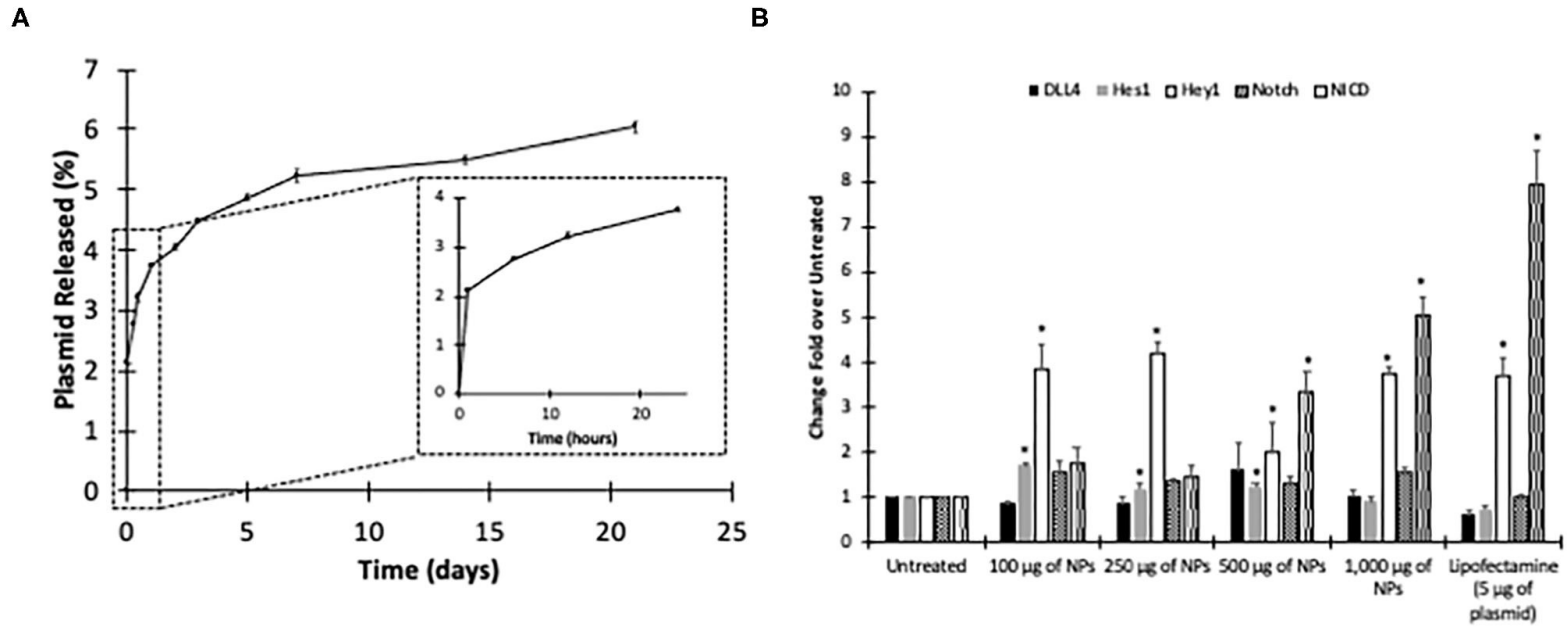
Characterization of NICD Loaded Nanoparticles. Release curve of **(A)** NICD Plasmid-loaded nanoparticles measured via Promega dsDNA assay after 21 days. *n* = 3. Data shown as mean ± standard deviation. **(B)** Quantitative expression of NICD after TetO-FUW-NICD Nanoparticle transfection at varying dosages. *Significantly different from Untreated Group evaluated via two-way ANOVA (*p* < 0.05).

Both pCAG-GFP and TetO-FUW-NICD were loaded into HMW PLGA nanoparticles at 62.3 ± 2.2 μg plasmid per mg of nanoparticles and 89.1 ± 6.4 μg plasmid/mg nanoparticles, respectively. The encapsulation efficiency of 56.3 ± 4.1 and 38.9 ± 2.17% for NICD and GFP plasmids, respectfully, is similar to previous reports (58–60) (Table 3). Additionally, previously reported particles were larger (59) and the encapsulated plasmids were 6- to 2-times smaller in size (58–60) of our largest plasmid, at 10,671 bp, the genetic material encapsulated into the nanoparticles was released in a similar form to our model hydrophilic drug, Rh B (Figure 5A, Supplementary Figures 1, 3). The NICD plasmid released up to 1 μg of plasmid within the first 24 h. The plasmid continued to be released over 14 days to a total of 1.2 μg (Figure 5A). Our GFP plasmid loaded nanoparticles similarly released 0.5 μg of plasmid over 14 days (Supplementary Figure 3). With the addition of the plasmids, the zeta potential and size both increased, indicating a change. However, the polydispersity value was still low illustrating their homogeneous size.

**Table 3:** NICD plasmid loaded PLGA nanoparticle characteristics.

	**Size (nm)**	**Poly dispersity**	**Zeta potential (mV)**	**Encapsulated efficiency (%)**
NICD-loaded PLGA nanoparticle	272 ± 51	0.12 ± 0.05	−12.9 ± 1.90	56.3 ± 4.1%
NICD-loaded PLGA nanoparticle conjugated with anti-Tie2+Tie1	268 ± 26	0.11 ± 0.01	−17.0 ± 0.83	

TetO-FUW-NICD loaded nanoparticles were also given to HUVECs at varying doses. Compared to the untreated group, Notch target gene, *Hey1* was upregulated in each tested concentration. Additionally, another target gene, *Hes1*, was upregulated with NICD plasmid concentrations of 100, 250, and 500 μg of nanoparticles while 1,000 μg of NICD plasmid loaded nanoparticle decrease the expression level of *Hey1* (Figure 5B). Based on the trend, the NICD plasmid concentration of nanoparticle, affects the gradual expression level of target gene expressions until adding 250 ug of NICD plasmid loaded NP.

### GFP Expression Over Time

**Figure 6:**
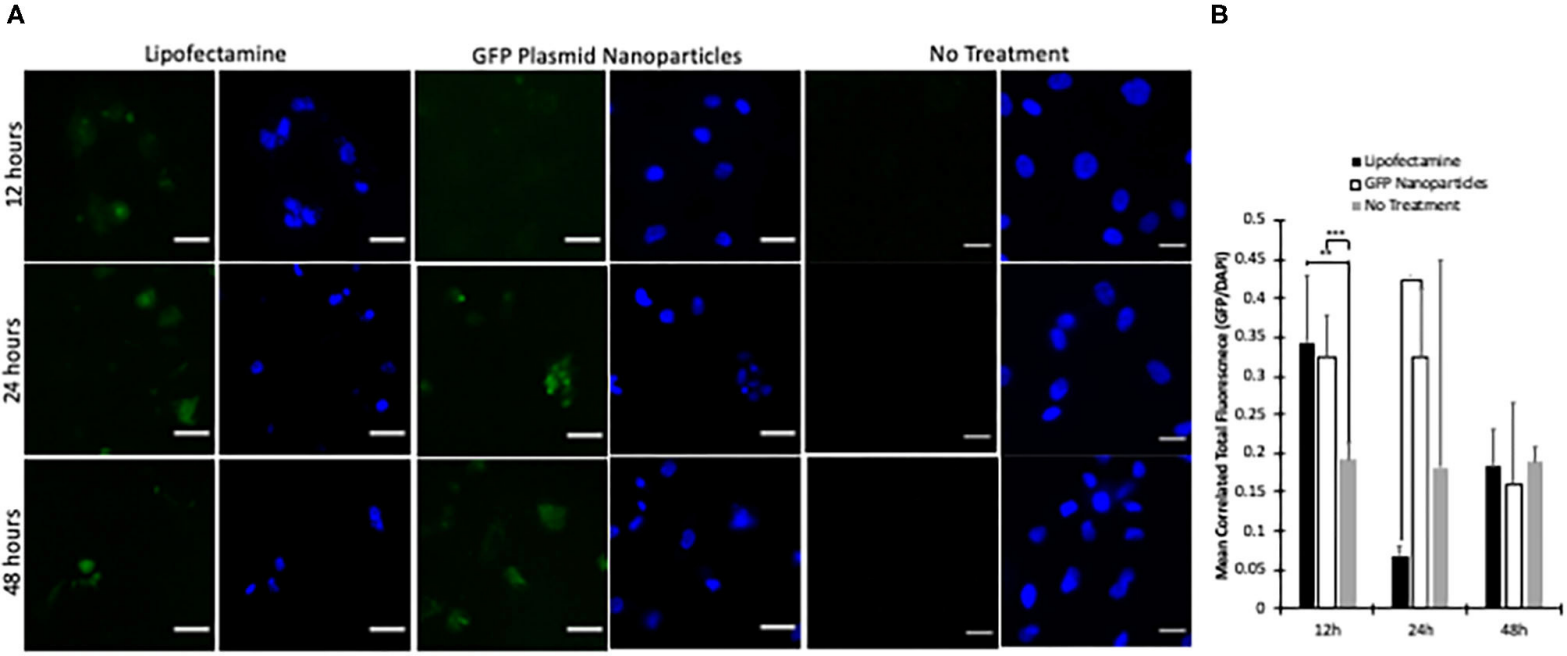
GFP plasmid-loaded nanoparticle transfection. **(A)** After 6 h of treatment, nanoparticles or lipofectamine were washed off with 1X PBS and media replaced. At 12 h, slight GFP expression can be seen (**A**, first row). After 24 h, there is significantly more GFP expression in GFP plasmid-loaded nanoparticles than lipofectamine treated cells (**A**, second row). Additionally, after 48 h, lipofectamine transfected cells had a high GFP signal in few cells. Whereas, nanoparticle transfected cells had many cells expressing GFP, resulting in a lower over signal. **(B)** Normalized GFP intensity to nuclei intensity shows significantly higher lipofectamine at 12 h, significantly higher Nanoparticle at 24 h, and no difference at 48 h. ∙ indicates (*p* < 0.1), **indicates (*p* < 0.01) and ***indicates significance at (*p* < 0.001) via one-way ANOVA per time point.

HUVECs were subjected to 5 μg of plasmid, either through Lipofectamine 3000, or our GFP Plasmid-loaded nanoparticles. After 6 h, the treatments were removed, and fresh media applied to the cells. At 12 h, Lipofectamine had significantly higher GFP expression than the plasmid nanoparticles (Figure 6B). However, after 24 h, the GFP plasmid loaded nanoparticles had a significantly higher GFP expression level per cell. Additionally, the GFP plasmid-loaded nanoparticles had an even expression of GFP across most cells. At 24 h, the lipofectamine group had few GFP positive cells compared to that of the nanoparticle treated group. At 48 h post transfection, GFP was observed in both lipofectamine treated and GFP plasmid-loaded nanoparticle treated groups (Figure 6).

### Nanoparticle Mediated HUVEC Transfection Based on NICD Expression

**Figure 7:**
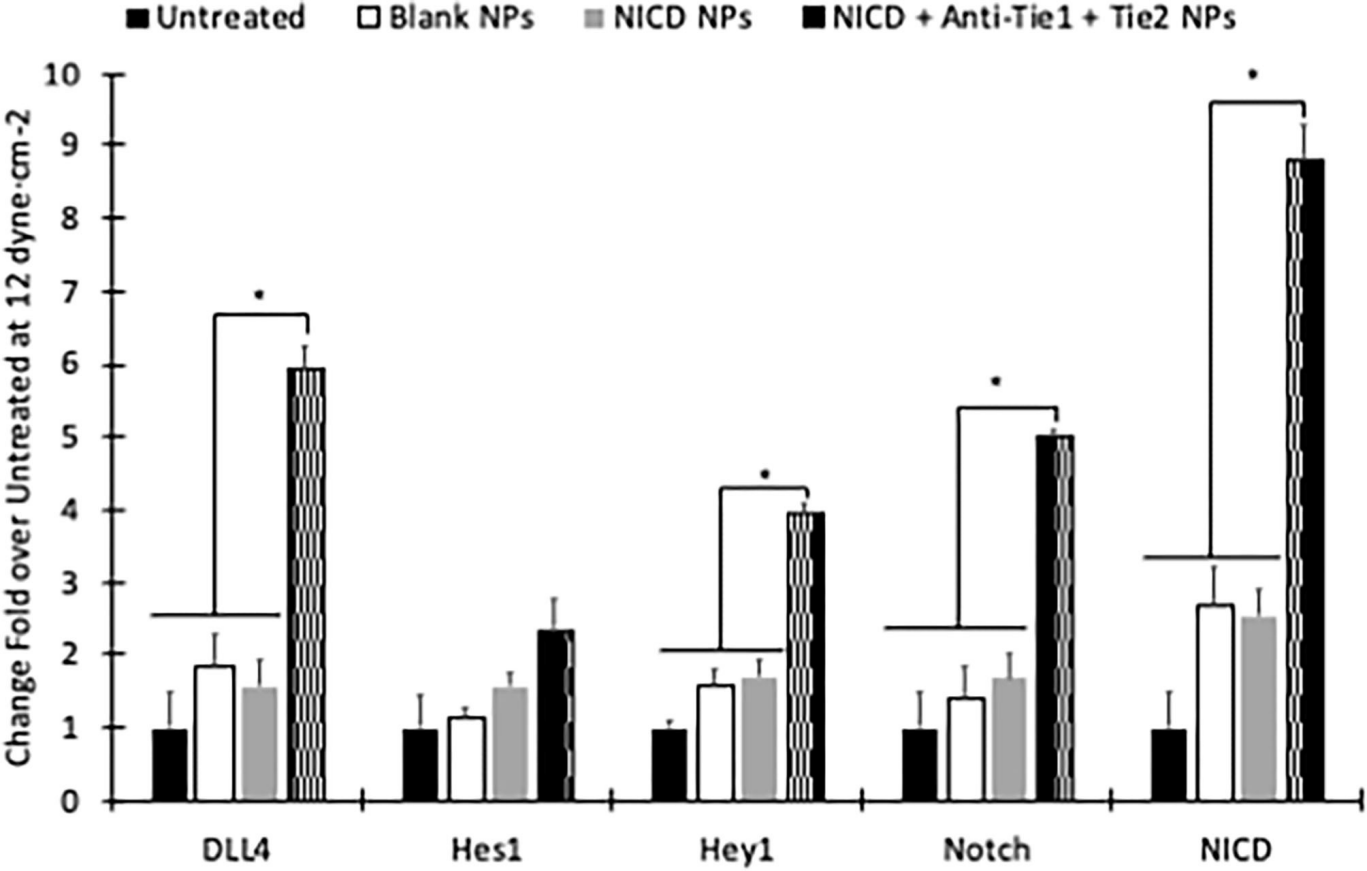
NICD plasmid-loaded nanoparticles can enhance NICD expression in dynamic culture conditions. RT-PCR results showing that NPs conjugated to anti-Tie2+Tie1 significantly upregulate DLL4, Hey1, Notch Receptor, and NICD in 12 dyne·cm^−2^ flow conditions. *NICD+Anti-Tie1+Tie2 is significantly higher mRNA expression evaluated with one-way ANOVA per gene group (*p* < 0.05).

HUVECs were subjected to 12 dyne·cm^−2^ for 24 h, then an additional 24 h of flow treatment with blank nanoparticles, TetO-FUW-NICD loaded HMW nanoparticles, TetO-FUW-NICD loaded HMW nanoparticles conjugated with anti-Tie2+Tie1, or cell media only for control. Each nanoparticle group was given at a concentration of 250 μg/mL due to highest Notch target efficiency concentration (Figure 5B). The plasmid-loaded nanoparticles with targeting antibody had significantly higher expression of Notch related genes, but not *Hes1* although expression level was upregulated (Figure 7). The expression of Notch related genes when exposed to plasmid-loaded nanoparticles without a conjugating antibody were not significantly different from that of the blank nanoparticles. Both were upregulated most likely due to the increased viscosity of the media after adding the nanoparticles. The higher viscosity causes a higher shear stress, which upregulates shear responsive Notch signaling Supplementary Figure 5. We then analyzed the protein expression after application of the NICD loaded nanoparticles, NICD loaded nanoparticles conjugated with anti-Tie2+Tie1, or no treatment. After 24 h of treatment, we found that the nanoparticles containing NICD plasmid and conjugated with anti-Tie2+Tie1 had a significantly higher amount of NICD protein than both the NICD-loaded nanoparticles and the no treatment group. This indicates that the plasmid was able to be released from the nanoparticle and be translated into protein by the cell Supplementary Figure 6. Additionally, the NICD-loaded nanoparticles had significantly higher Hes1 and Hey1 proteins (Supplementary Figure 6).

## Discussion

In this work, we have demonstrated the successful transfection of NICD plasmid to upregulated Notch signaling via PLGA nanoparticles. PLGA is one of the most characterized biopolymers with respect to drug delivery design and performance (61), which has been widely utilized for delivering proteins (51, 62–64) and hydrophobic drugs (65–68). More recently, PLGA nanoparticle has been used as a delivery vehicles for gene delivery for vaccines (32), immunotherapy (29), or gene therapy (38, 58, 60, 69, 70). Therefore, we have used PLGA for NICD DNA plasmid delivery to overcome the limitations of traditional viral vector methods such as negative immunological effects, random gene integration, base pair size restrictions, and cytotoxicity (71).

First, we optimized the molecular weight of the PLGA. Our data shows that the higher molecular weight, 55–65 kDa, PLGA was more cytocompatible, hemocompatible, and stable in various solutions. Even though the low molecular weight, 5–10 kDa, released the plasmid quickly, the nanoparticles were unstable in saline, a common liquid vehicle used for intravenous drug delivery (68, 72–75). Similarly, it has been shown that low molecular weight PLGA nanoparticles release the payload at a higher rate (76–78). Therefore, the molecular weight can influence how long a drug of interest is released and exposed to the area of interest (77, 78). These previous studies support our data showing that the low molecular weight releases the payload after 5 days. Interestingly, molecular weight differences of 5 kDa can have significantly different release profiles (76). Additionally, lower molecular weight PLGA increases the pH of the surrounding fluid, which leads to cell death (79). This would explain why at high concentrations, our lower molecular weight PLGA nanoparticles become significantly more toxic (Figure 2C). Mittal et al. showed that the poly-lactic acid to poly-glycolic acid ratio (PLA: PGA) have a significant effect of drug release as well (78). Mittal et al. shows that the 50:50 composition allows for the highest release of the payload compared to 65:35 and 85:15 ratios (78). They also show that *in vivo*, the higher molecular weight polymers allow for a higher cumulative drug in the blood stream in both oral and intravenous administration (78). Additionally, others have shown that PLGA (50:50, 24–38 kDa) is non-toxic to cells with survival rates >90%, and hemolysis of <0.4% (80). Our results support that of Thasneem et al. with cell viability of >90% at all tested concentrations for high molecular weight PLGA. We expanded that other molecular weights of PLGA, that are 10 × lower and 2 × higher than Thasneem's, have <0.3% hemolysis at all tested concentrations. Combining our data with those mentioned, PLGA is shown to be non-toxic, hemocompatible, and stable. Specifically, we show that higher molecular weight PLGA has superior performance over that of the low molecular weight PLGA, therefore, we have chosen the high molecular weight PLGA nanoparticles for antibody optimization.

As intravenous injection is the most common method to administer therapeutics, it is critical to ensure that the nanoparticle reaches its targeted destination. Although research reported that encapsulated DNA into particles have modified their nanoparticles to be less toxic, have higher cellular uptake, or increase payload (39, 41, 81), there still is the limitation of off target delivery which causes systemic effects (82, 83). For this reason, we investigated two endothelial cell specific antibodies, anti-EGFL7 and anti-Tie2+Tie1, on their ability to enhance cellular uptake in static and dynamic environments. We show that anti-Tie2+Tie1 has superior cellular uptake in both static and dynamic cell culture environments (Figures 4, 6). Additionally, we have demonstrated that compared to HL1 cells, there was significantly more cellular uptake of anti-Tie2+Tie1 conjugated nanoparticles in HUVECs, which is due to the fact that HL1 cells do not express Tie2 or Tie1 proteins (84) (Supplementary Figure 4C). With our binding study, the anti-Tie2+Tie1 conjugated nanoparticles were bound significantly more than BSA conjugated or unconjugated nanoparticles. Additionally, the anti-Tie2+Tie1 nanoparticles bound significantly more to the Tie1+Tie2 coated slides than BSA-coated or uncoated slides (Supplementary Figures 4A,B). Others have shown that even in co-cultures of MCF-10A neoT and Caco-2, targeting antibodies for ductal breast carcinoma selectively target the MCF-10A neoT cells (85). Unconjugated nanoparticles were up taken by both cell types in the co-culture (85). Other targeting nanoparticles have been able to repress expression of particular genes at a higher rate than the standard (75). Compared to unconjugated nanoparticles, our targeting nanoparticles had significantly higher cellular uptake in the dynamic culture, supporting the notion that without targeting, the therapeutic clearance may diffuse the efficacy of the therapeutic.

In addition to site specific delivery, the encapsulated DNA needs to be bioactive. Others have shown that the sonication time or power, additives, or polymer molecular weight can affect the integrity of the plasmid (60). We have shown that our synthesis method ensures plasmid delivery at several nanoparticle concentrations, and that the plasmid is bioactive. To find optimum concentration of NICD to upregulate Notch signaling related genes, the 100 and 250 μg nanoparticle of NICD encapsulating nanoparticles significantly upregulated Notch target genes, *Hes1* and *Hey1*, compared to the gold standard lipofectamine with 5 μg of NICD (Figure 5B). We show that GFP protein can be synthesized in HUVECs by delivering the plasmid. Additionally, Notch and its related genes were quantified showing upregulation. However, in 500 and 1,000 μg nanoparticle groups, while NICD was also significantly upregulated, expression levels of target genes were downregulated. This indicates that the 100 μg or 250 μg of NICD nanoparticle concentrations were preferred to induce a downstream genetic effect although the higher concentrations were able to increase expression of NICD. Accordance with previous report, increment of NICD does not proportionally increase target gene expression levels (26).

Although we demonstrated PLGA nanoparticles at HMW (55–65 kDa) are an appropriate material to deliver NICD plasmid to upregulate Notch signaling with *in vitro* flow experiment, we still need to evaluate our nanoparticle in an *in vivo* environment. Specifically, our *in vitro* experiment was limited in laminar flow, while *in vivo* injection of nanoparticle would be exposed to pulsatile blood flow environment. In addition, our optimal endothelial targeting antibody, anti-Tie2+Tie1, for this study may bind to only activated Tie2 and Tie1 proteins when phosphorylated during vasculogenesis and vessel maturation (86, 87). Although our antibodies target toward to Tie1 and Tie2 heterodimer after activation from shear stress application (86), application of conjugated PLGA nanoparticles was mainly for fluid shear studies to enhance the targeting ability toward endothelial cells; thus, our nanoparticles could successfully target endothelial cells in this study. In future studies, we will optimize the NICD plasmid concentration, and the anti-Tie2+Tie1 concentration for conjugation to PLGA nanoparticles for upregulated Notch signaling in an animal model. This future experiment will help to translate our technology to effective therapeutic approach for translational medicine.

In this study, we have synthesized a PLGA nanoparticle that can deliver NICD plasmids to primary endothelial cells to upregulate Notch related components. In addition to being a non-viral transfection agent, the optimized nanoparticle was compatible with human cells and blood, and effectively delivered bioactive plasmid DNA to endothelial cells. These results demonstrate that PLGA targeting nanoparticles could increase the genetic delivery in complex environments, such as *in vivo*, with minimal adverse effects.

## Conclusion

In this work, we have shown that higher molecular weight PLGA outperforms the low molecular weight PLGA nanoparticles in cytotoxicity, cellular uptake, stability, and hemocompatibility. Additionally, the conjugation of anti-Tie2+Tie1 to the nanoparticles allows for a significant increase in endocytosis compared to those conjugated with anti-EGFL7. Lastly, our pCAG-GFP and TetO-FUW-NICD plasmids were both successfully encapsulated and transfected into HUVECs. Most importantly, the plasmid was bioactive after transfection as indicated by GFP imaging and RT-PCR analysis. In conclusion, we can show that plasmid loaded nanoparticles have a higher transfection efficiency and create a significant genetic effect when applied to hard-to-transfect cells like HUVECs.

## Data Availability Statement

The datasets presented in this study can be found in online repositories. The names of the repository/repositories and accession number(s) can be found below: https://www.addgene.org/61540/. https://www.addgene.org/11150/.

## Ethics Statement

The studies involving human participants were reviewed and approved by the University of Texas at Arlington Institutional Biosafety Committee (IBC 18.001). The patients/participants provided their written informed consent to participate in this study.

## Author Contributions

VM and AK: nanoparticle characterization, hemocompatibility, and antibody optimization. VM and UC: plasmid loading, plasmid release analysis, and flow study RT-PCR. VM, UC, and TD: HUVEC cell culture. VM and SL: nanoparticle release study. VM, SL, and LK: bacteria culture, and plasmid isolation. JJ, ZP, HS, KN, and JL: project support, guide, and financial support. All authors contributed to the article and approved the submitted version.

## Funding

VM and AK are supported by the National Institutes of Health (NIH) training award, T32 HL134613 (KN). JL is supported by the American Heart Association 18CDA34110150 (JL) and NSF 1936519 (JL).

## Conflict of Interest

The authors declare that the research was conducted in the absence of any commercial or financial relationships that could be construed as a potential conflict of interest.

## Publisher's Note

All claims expressed in this article are solely those of the authors and do not necessarily represent those of their affiliated organizations, or those of the publisher, the editors and the reviewers. Any product that may be evaluated in this article, or claim that may be made by its manufacturer, is not guaranteed or endorsed by the publisher.
