# Corrigendum: Notch Intracellular Domain Plasmid Delivery via Poly(Lactic-Co-Glycolic Acid) Nanoparticles to Upregulate Notch Pathway Molecules

**DOI:** 10.3389/fcvm.2021.785910

**Published:** 2021-10-26

**Authors:** Victoria L. Messerschmidt, Uday Chintapula, Aneetta E. Kuriakose, Samantha Laboy, Thuy Thi Dang Truong, LeNaiya A. Kydd, Justyn Jaworski, Zui Pan, Hesham Sadek, Kytai T. Nguyen, Juhyun Lee

**Affiliations:** ^1^Department of Bioengineering, University of Texas at Arlington, Arlington, TX, United States; ^2^Department of Internal Medicine, University of Texas Southwestern Medical Center, Dallas, TX, United States; ^3^College of Nursing and Health Innovation, University of Texas at Arlington, Arlington, TX, United States

**Keywords:** Notch signaling, PLGA, nanoparticles, gene delivery, non-viral transfection

An author name was incorrectly spelled as ^**^Hashem Sadek^**^. The correct spelling is ^**^Hesham Sadek^**^.

^**^ **Incorrect version** ^**^

Victoria L. Messerschmidt^1,2†^, Uday Chintapula^1,2†^, Aneetta E. Kuriakose^1,2^, Samantha Laboy^1^, Thuy Thi Dang Truong^1^, LeNaiya A. Kydd^1^, Justyn Jaworski^1^, Zui Pan^3^, Hashem Sadek^2^, Kytai T. Nguyen^1,2*^ and Juhyun Lee^1,2*^

^1^ Department of Bioengineering, University of Texas at Arlington, Arlington, TX, United States

^2^ Department of Internal Medicine, University of Texas Southwestern Medical Center, Dallas, TX, United States

^3^ College of Nursing and Health Innovation, University of Texas at Arlington, Arlington, TX, United States

^**^ **CORRECT version** ^**^

Victoria L. Messerschmidt^1,2†^, Uday Chintapula^1,2†^, Aneetta E. Kuriakose^1,2^, Samantha Laboy^1^, Thuy Thi Dang Truong^1^, LeNaiya A. Kydd^1^, Justyn Jaworski^1^, Zui Pan^3^, Hesham Sadek^2^, Kytai T. Nguyen^1,2*^ and Juhyun Lee^1,2*^

^1^ Department of Bioengineering, University of Texas at Arlington, Arlington, TX, United States

^2^ Department of Internal Medicine, University of Texas Southwestern Medical Center, Dallas, TX, United States

^3^ College of Nursing and Health Innovation, University of Texas at Arlington, Arlington, TX, United States

The authors apologize for this error and state that this does not change the scientific conclusions of the article in any way. The original article has been updated.

## Publisher's Note

All claims expressed in this article are solely those of the authors and do not necessarily represent those of their affiliated organizations, or those of the publisher, the editors and the reviewers. Any product that may be evaluated in this article, or claim that may be made by its manufacturer, is not guaranteed or endorsed by the publisher.

