# Supplementary material for: Notch Intracellular Domain Plasmid Delivery via Poly(Lactic-Co-Glycolic Acid) Nanoparticles to Upregulate Notch Pathway Molecules
Source: Front Cardiovasc Med. 2021 Sep 28;8:707897. doi: 10.3389/fcvm.2021.707897 (PMC8507495; doi:10.3389/fcvm.2021.707897)
Supplement: Supplementary file 1 [file Data_Sheet_1.PDF]

Notch Intracellular Domain Plasmid Delivery via Poly(lactic-co-glycolic acid) Nanoparticles to Upregulate Notch Signaling

Supplemental Data

Victoria L. Messerschmidt[1,2†], Uday Chintapula[1,2†], Aneetta E. Kuriakose[1,2], Samantha Laboy[1], Thuy Thi Dang Truong[1], LeNaiya A. Kydd[1], Justyn Jaworski[1], Zui Pan[3], Hashem Sadek[2], Kytai T. Nguyen[1,2*], Juhyun Lee[1,2*]

[1]Department of Bioengineering, University of Texas at Arlington, Arlington TX 76010 USA

[2]University of Texas Southwestern Medical Center, Dallas TX 75390 USA

[3]College of Nursing and Health Innovation, University of Texas at Arlington, Arlington TX 76010 USA

[†] **These authors have contributed equally to this work**

Supplementary Figures

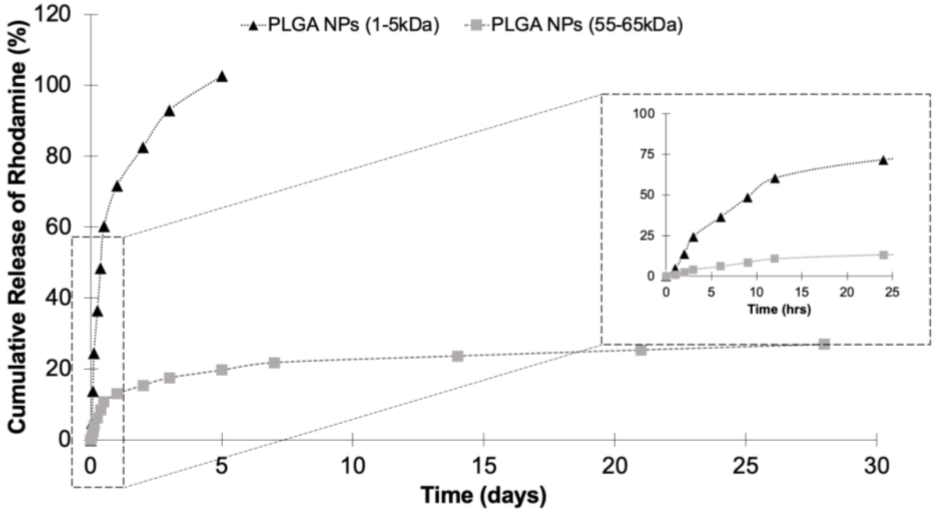

Supplementary Figure 1: Rhodamine Release from High and Low Molecular Weight PLGA Nanoparticles. *Release of Rhodamine into the supernatant has a burst release, followed by a sustained release up to 28 days. The Low Molecular Weight nanoparticles release 100% of loaded rhodamine by 5 days. Inset shows initial burst release up to 24 hours.*

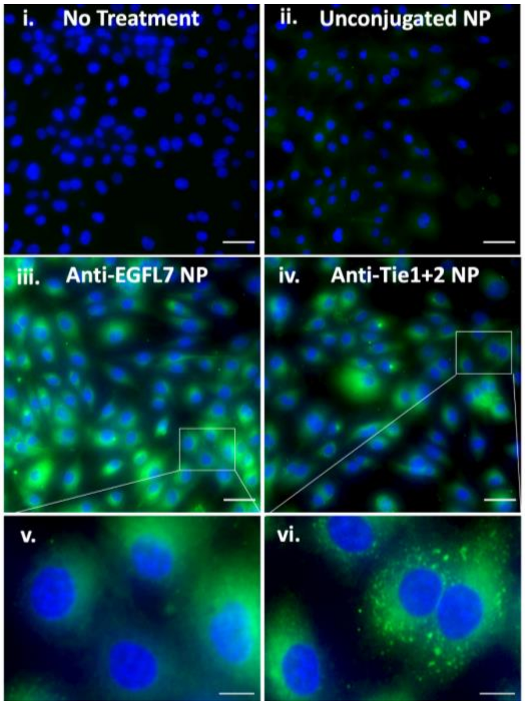

Supplementary Figure 2: Static Culture of Antibody Conjugated Nanoparticles. *HUVEC's cultured with media only (i.), unconjugated nanoparticles (ii.), anti-EGFL7 conjugated nanoparticles (iii.), or anti-Tie2+Tie1 conjugated nanoparticles (iv.). Scale bar = 20 μm. Higher magnification of HUVEC's cultured in anti-EGFL7 conjugated nanoparticles (v.) or anti-Tie2+Tie1 conjugated nanoparticles. Scale bar = 5 μm.*

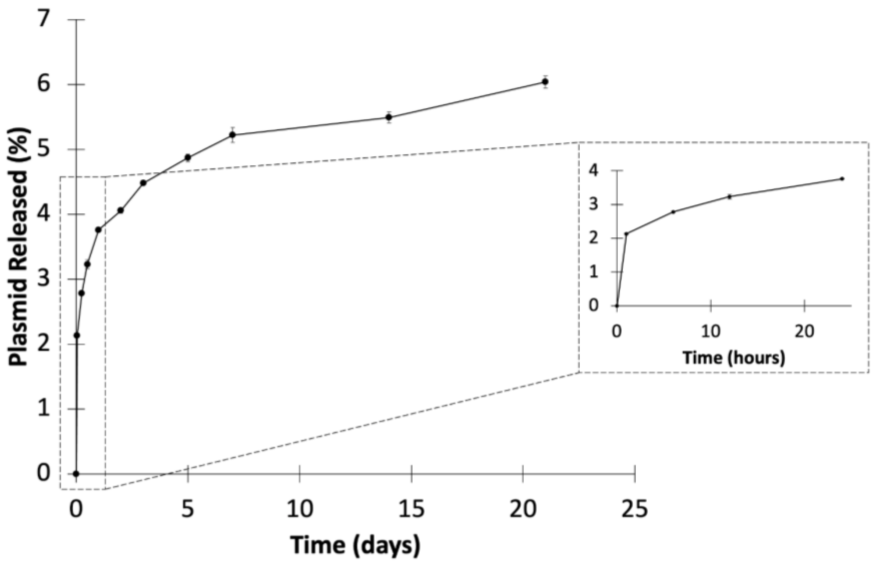

Supplementary Figure 3: Characterization of GFP Plasmid-Loaded PLGA Nanoparticles. *GFP plasmid released from HMW PLGA nanoparticles over 21 days. Inset is detailed graph of the first 24 hours of plasmid release. Data is shown as mean ± standard deviation.*

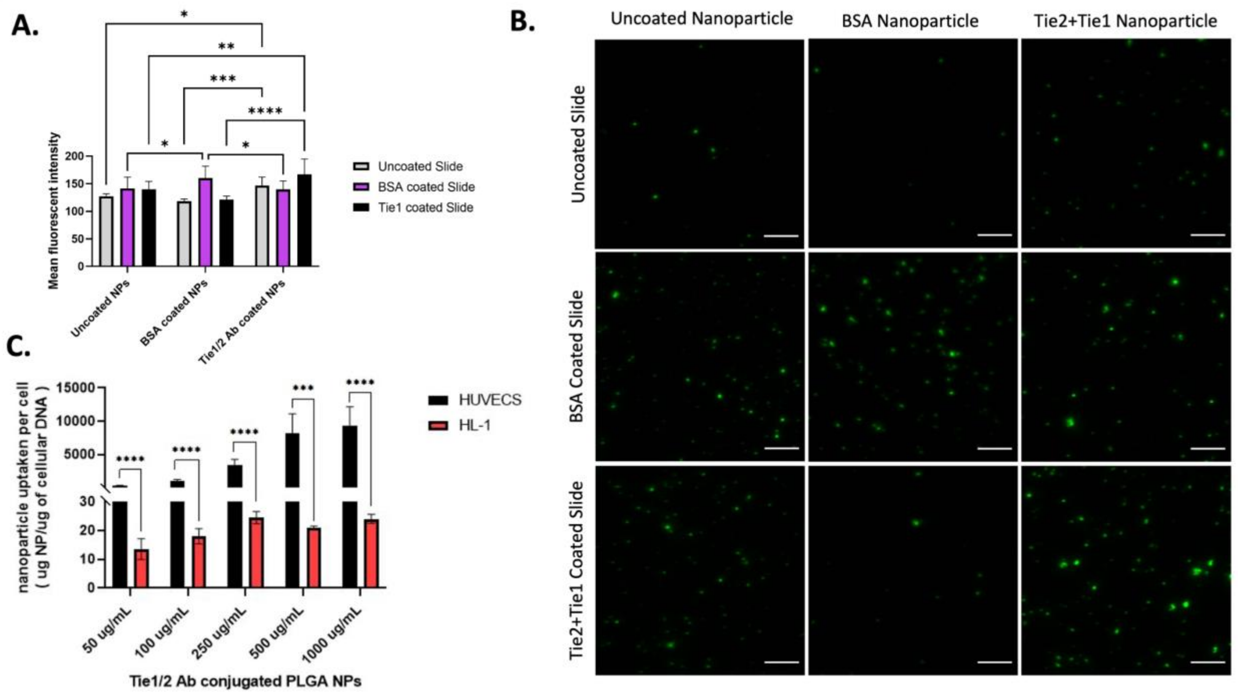

Supplementary Figure 4: Antibody Targeting Specificity. *(A) Quantitative evaluation of protein coated nanoparticles (BSA, Tie2+Tie1, or uncoated NPs) flowed over protein coated slides (BSA, Tie2+Tie1, or uncoated slides) at 5 dyne·cm^{-2}. * indicates ($p < 0.05$), ** indicates ($p < 0.01$), *** indicates ($p < 0.001$), **** indicates ($p < 0.0001$) evaluated using a two-way ANOVA.(B) Qualitative images of coumarin-6 nanoparticles with differing protein coatings. Scale bar = 5 μm. (C) Cellular uptake analysis of anti-Tie2+Tie1 conjugated nanoparticles cultured with HUVECs and HL1 cells. *** indicates ($p < 0.001$), **** indicates ($p < 0.0001$) evaluated via t-test per concentration. All data shown as mean + standard deviation.*

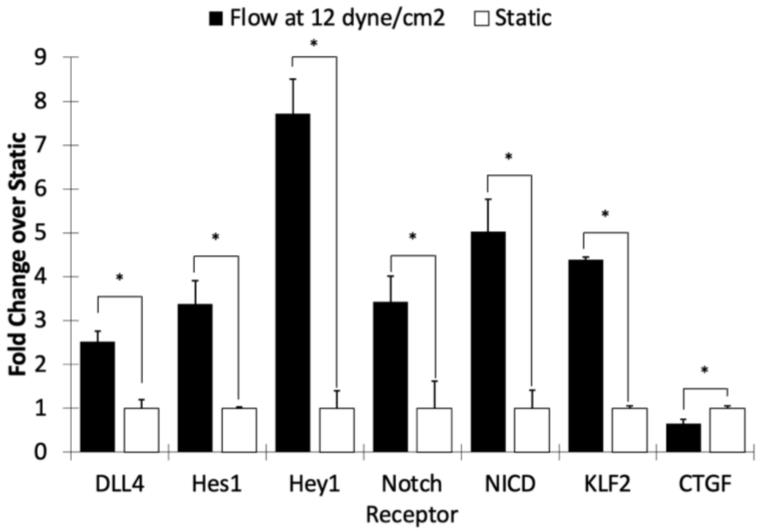

Supplementary Figure 5: Natural Upregulation of Notch Related Genes due to Shear Stress. *RT-PCR results showing that notch related genes are upregulated from shear stress stimulus. KLF2 is also upregulated due to shear stress, while CTGF is downregulated.*

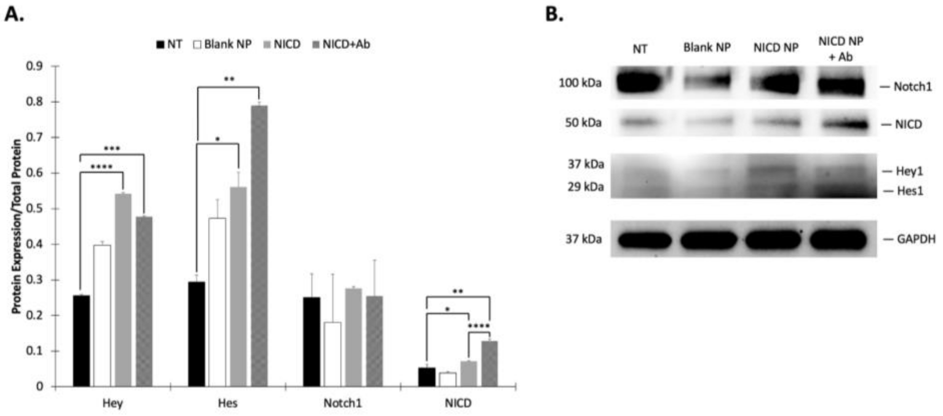

Supplementary Figure 6: Western Blot after Nanoparticle Treatment with shear stress application. (A) *Evaluation of protein content after treatment with NICD loaded nanoparticles, NICD loaded nanoparticles with anti-Tie2+Tie1 conjugated, blank nanoparticles, or cell media only and 12 dyne·cm*$^{-2}$ *of shear. Data is shown as mean ± standard deviation. **** indicates (p < 0.0001), *** indicates (p < 0.001), ** indicates (p < 0.01), and * indicates group is significantly different (p < 0.05) evaluated using one-way ANOVA. (B) Western blot analysis showing upregulation of NICD plasmid upon delivery with nanoparticles conjugated with anti-Tie2+Tie1 for 24 hours in HUVECs. NT: No treatment, NP: nanoparticle, NICD: Notch intracellular domain.*

Supplementary Tables

Supplementary Table 1: *Physical Characteristics of GFP Plasmid-Loaded PLGA Nanoparticles.*

	**Size (nm)**	**Poly Dispersity**	**Zeta Potential (mV)**	**Encapsulation Efficiency (%)**
**GFP Plasmid-Loaded PLGA Nanoparticles**	278.6 ± 47	0.22 ± 0.05	-14.8 ± 2.0	38.9 ± 2.17 %
